# Preliminary data on a mnemonic instrument with proverbs for tracking Alzheimer’s disease

**DOI:** 10.1590/S1980-57642009DN20400018

**Published:** 2008

**Authors:** Mauricéa Tabósa Ferreira Santos, Gutemberg Guerra, Terce Liana de Menezes, Tatiana Lins Carvalho, João Carlos Alchieri, Everton Botelho Sougey

**Affiliations:** 1 Occupational Therapist, gerontologist and doctorate student in the Postgraduate Program of Health Sciences (UFRN).; 2 Neurologist, Master in Neuropsychiatry and coordinator of Behavioral and Cognitive Neurology Outpatient Facility (UFPE).; 3 Neurologist, Doctor of Neuropsychiatry and head of the Cognitive Neurology Outpatient Facility of the Center for Elderly Care (UFPE).; 4 Occupational therapist at the Outpatient Facility of Oswaldo Cruz Hospital and specialist in Gerontology at the Faculdade Integrada do Recife (FIR).; 5 Psychologist, Doctor of Psychology and adjunct professor II in the Department of Psychology of UFRN.; 6 Associate Professor, PhD, Department of Neuropsychiatry of UFPE.

**Keywords:** memory, executive function, understanding proverbs, Alzheimer’s disease

## Abstract

In the differential diagnosis between Alzheimer’s disease (AD) and normal aging, tests evaluating memory and executive functions are frequently used. The addition of abstraction tests may enhance the effectiveness of screening tests for AD.

**Objectives:**

The aim of this study was to compare initial data of a new instrument – The Screening Test for Alzheimer’s Disease with Proverbs (STADP) – against other screening tests used in AD diagnosis.

**Methods:**

Sixty elderly individuals (46 controls and 14 AD subjects with CDR=1), aged =60 years, with at least one year of schooling, were evaluated using the STADP at outpatient clinic. The STADP assesses short-term memory, episodic memory, executive functions and language, in addition to proverb recognition. The performance of the participants on the Mini-Mental State Examination (MMSE), semantic Verbal Fluency (VF) and Clock Drawing Test (CDT) were evaluated and the habit of reading, writing and sociodemographic data were also taken into account.

**Results:**

There were significant correlations between STADP and the performance on the MMSE (r=0.64), CDT (r=0.50) and VF (r=0.56). Age influenced all sub-items of the STADP, specifically episodic memory (r= –0.54), whereas schooling mainly influenced executive functions and language (r=0.46). The total score, stages A and C and the “proverb recognition” of STADP (p<0.001), as well as the MMSE (p<0.001), CDT (p=0.016), VF (p<0.001) were significantly different in AD versus control groups.

**Conclusions:**

The findings point to the potential use of the STADP in AD, warranting the conducting of further studies.

## Abstract (pt)

No diagnóstico diferencial entre a doença de Alzheimer (DA) e envelhecimento normal testes que avaliam memória e função executiva são freqüentemente utilizados. A adição de testes de abstração ao de rastreio pode ampliar ainda mais a eficácia de testes de rastreio para DA.

**Objetivos:**

O objetivo deste trabalho foi comparar dados iniciais do novo instrumento – Teste de Rastreio de Doença de Alzheimer com Provérbios (TRDAP) – com testes padronizados para diagnóstico de DA.

**Métodos:**

Sessenta idosos (46 controles e 14 DA com CDR=1), escolaridade mínima de um ano, a partir de 60 anos, atendidos em ambulatórios foram avaliados com o TRDAP. O TRDAP avalia memória de curto prazo, memória episódica e funções executivas e linguagem, além do reconhecimento de provérbios. Foram usados o Mini-Exame Mental (MEEM), a Fluência Verbal semântica (FV), o Teste do Desenho do Relógio (TDR) e observados hábitos de leitura, escrita e dados sócio-demográficos.

**Resultados:**

Houve significativa correlação entre: TRDAP e MEEM (r=0,64), TDR (r=0,50) e FV (r=0,56). Idade influenciou os três testes, especificamente memória episódica do TRDAP (r= –0,54); escolaridade, em funções executivas e linguagem (r=0,46). Na comparação entre pacientes com DA e controles o escore total e as etapas A e C do TRDAP e o reconhecimento dos provérbios (p<0,001), assim como o MEEM (p<0,001), o TDR (p=0,016) e FV (p<0,001).

**Conclusões:**

Os achados apontam para utilidade potencial do TRDAP na DA, indicando que mais estudos devem ser realizados.

The devising of diagnostic methods to identify and prognosticate new cases of Alzheimer’s disease (AD) more swiftly is an emergency of socioeconomic importance. The search for clinical markers in the early stage of AD points to a significantly lower percentage of hits than that of the control group in episodic memory tests (more so in verbal memory) and short-term or working memory. This explains the low performance seen in other cognitive tasks, in addition to significantly longer response latencies in all the tests, indicating a slowing of the information processing by the central nervous system in AD^1^ With regard to language, Mansur et al.^2^ reported that this is difficult to analyze in AD because of the intricate way in which it manifests itself in the functions of memory and attention, and given that working memory – a system that temporarily stores and manipulates the information needed for complex cognitive functions^3^ – is involved in many language processes, such as sentence and text comprehension. Longitudinal studies analyzing the integration of the operational aspects of the attention network with semantic aspects are essential. These studies should also focus on the relationship of the semantic domain, which is linked to the capacity to retain general facts and knowledge about the world,^4^ with the processing of lexical and discourse aspects, observing a mutual interference in the domain of lexical knowledge in the processes of understanding and recalling items. There is a need for early language diagnosis in AD, with assessments consisting of the oral comprehension of texts that analyze the different memory subsystems (short and long term). According to Jacobson et al.,^5^ executive function deficits occur as a result of the dementia processes and may be used as differential diagnostic markers between dementias and normal aging; Hamdan and Bueno^6^ also found significant differences in relation to tests of executive control in control groups and patients with initial AD. Moreover, other authors have suggested the use of executive function tests in the initial phase of AD.^7,8^ Therefore, the addition of abstraction tests to those of screening tools may enhance the effectiveness of instruments such as the Mini-Mental State Examination (MMSE),^9^ and the Clock Drawing Test (CDT), cited in the current literature.^10-12^ Furthermore, tests of abstraction using proverbs may prove valuable test,^13^ and have already been used in literature since the 1950s.^14-16^ According to Siviero,^17^ the interpretation of proverbs involves various cognitive aspects: declarative memory, concept categorization, analogies between figures and words (temporal and parietal lobes), logic reasoning (frontal lobe) and verbalizing understanding (left temporal lobe and motor areas).

In this article we present the preliminary profile data of patients with early AD and of control individuals on the Screening Test for Alzheimer’s Disease using Proverbs (STADP).

## Methods

Sixty elderly individuals (46 control individuals and 14 patients with AD), aged ≥60 years, 26% men, with at least one year of schooling and who were able to read, were evaluated at the Cognitive and Neurology Ambulatory Facility of the Center for Elderly Care and at the Behavioral and Cognitive Neurology Ambulatory Facility, Open University for the Third Age (UNATI), all at Federal University of Pernambuco, or at the Elderly Care Program of the Areias General Hospital (HGA), specialized in treating Alzheimer’s or at a private geriatric clinic.

The diagnoses of dementia were performed by neurologists and geriatricians according to DSM-IV criteria, using the MMSE and the Clinical Dementia Rating (CDR). All patients with AD were taking cholinesterase inhibitor and/or memantine. Only AD patients with CDR=1 and with scores of at least 19 on the MMSE were included. The control participants were all CDR=0 and scored at least 25 on the MMSE. Data collection took place in a quiet climate-controlled room. Interviews were conducted to obtain socioeconomic data and information on a number of habits.^18^ The following instruments were used: the STADP – that assesses memory, executive function and language, the MMSE – an assessment of overall cognition, the semantic verbal fluency (VF) for language and executive functions, and the spontaneous CDT – an evaluation of executive functions.

**Figure 1:**
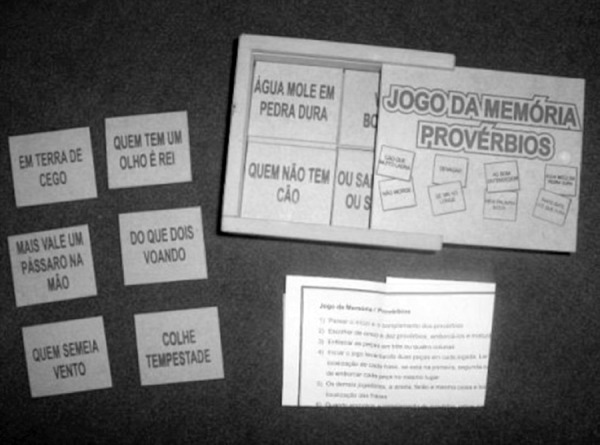
The memory game of proverbs.

The STADP contains six pairs of wooden pieces from the Memory Game of Proverbs (Figure 1),^19^ with legible letters and six proverbs divided into two parts (initial and final). There are three stages (A, B, C) as follows: “A” consists of soliciting the pairing and memorization of the first three proverbs, reading them out loud and then turning them face down; “B” involves pairing three others and interpreting them; these are read by the researcher, who records the interpretation; and “C” the recall of the first three proverbs. The total score is 15 points, subdivided into three: (A), 6 points for short-term memory with three consecutive attempts and possibility of learning; (B), 3 points for executive functions – abstraction/language-syntax; and (C), 6 points for delayed recall. The result is the sum of the three scores. At the end, six proverbs were shown containing the first three (A) for recognition.

The study was approved by the Research Ethics Committee of the Federal University of Pernambuco on 22 May, 2007 (protocol number 072/07). An informed consent form was signed by all the participants and witnessed by a family member, when the former were accompanied. For statistical analysis we used the Mann-Whitney U test to compare the sociodemographic data and the performance on the tests by control subjects and AD patients. Pearson’s correlation coefficients (r) were used to analyze the correlations between the variables age, schooling, stages scores (A, B, C) on the STADP, MMSE, semantic VF and CDT, while Spearman’s correlation coefficient (rho) was used for correlation with habits. The SPSS software, version 15.01, was used for the statistical analysis.

## Results

The means and standard deviations of age were 70.1 years (7.2), and of schooling years was 9.2 years (4.2). Table 1 shows means and standard deviations of the performance on the MMSE, semantic VF and CDT and on STADP of the patients and control individuals.

**Table 1:** Demographic data and scores on tests among patients with Alzheimer's disease and control individuals.

Variables	Group	N	Means	SD	p
Age	Control	46	67.63	5.81	<0.001
	AD	14	76.93	5.92	
Schooling years	Control	46	9.48	4.13	0.979
	AD	14	9.57	5.00	
MMSE	Control	46	27.46	1.50	<0.001
	AD	14	21.86	2.88	
MMSE (recalled words)	Control	46	1.57	1.07	<0.001
	AD	14	0.21	0.80	
Spontaneous CDT	Control	46	12.26	2.60	0.016
	AD	14	8.93	4.45	
Semantic Verbal Fluency test (VF)	Control	46	16.20	4.32	<0.001
	AD	14	10.71	2.92	
STADP (score total)	Control	46	10.9	2.97	<0.001
	AD	14	15.46	2.48	
Short-term memory (stage A) STADP	Control	46	5.19	0.85	<0.001
	AD	14	3.67	1.08	
Executive functions / language (stage B) STADP	Control	46	1.78	1.15	0.073
	AD	14	1.14	1.17	
Episodic memory (stage C) STADP	Control	46	3.93	2.06	<0.001
	AD	14	0.64	1.34	
Proverb Recognition (STADP)	Control	46	2.96	0.21	<0.001
	AD	14	1.64	1.34	

STADP, Screening Test for Alzheimer's Disease using Proverbs; MMSE, Mini-Mental State Examination; CDT, Clock Drawing Test.

Age, the MMSE, semantic VF and CDT as well as the STADP (score total), short-term memory (stage A) STADP, episodic memory (stage C) STADP and the “proverb recognition” of STADP were significantly different between patients and controls. Conversely, schooling years and executive functions / language (stage B) of STADP did not differ, although there was a trend for significance in stage B of the STADP.

Spearman’s correlation coefficient showed correlation of schooling years with the habit of writing (rho=0.50) whereas the correlation with habit of reading was lower (rho=0.15).

There were inverse correlations between age with the STADP – total score, stages A and C, as well as with the “recognition of proverbs” and with the MMSE, semantic VF and CDT. With regard to the B stage (executive functions and language) there was no correlation with age, the opposite occurring with schooling years, for which stage B showed significant correlation (Table 2) (r=0.46).

**Table 2:** Correlation between variables and stage of Screening Test for Alzheimer's Disease using Proverbs.

	Age	Sch. years	STADP score total	STADP stage A	STADP stage B	STADP Stage C	MMSE	MMSE recorded words	CDT	VF	STADP proverb recogn.
Age	1	-0.08	-0.49**	-0.37**	-0.09	-0.54**	-0.57**	-0.45**	-0.39**	-0.41**	-0.48**
Sch. years		1	0.21	0.08	0.46**	0.07	0.06	0.29*	0.21	0.26*	0.03
Total STADP l			1	0.82**	0.59**	0.88**	0.64**	0.51**	0.50**	0.56**	0.38**
STADP stage A				1	0.48**	0.56**	0.52**	0.38**	0.51**	0.45**	0.40**
STADP stage B					1	0.21	0.28*	0.22	0.29*	0.47**	0.02
STADP stage C						1	0.62**	0.51**	0.40**	0.44**	0.40**
MMSE							1	0.62**	0.54**	0.57**	0.62**
MMSE recalled words								1	0.17	0.44**	0.43**
CDT									1	0.38**	0.41**
VF										1	0.19
STADP proverb recog.											1

CDT, Clock Drawing Spontaneous Test; MMSE, Mini-Mental State Examination; recog.: recognition; sch. Years: schooling years; STADP Screening Test for Alzheimer's Disease using Proverbs; STADP (stage A), Short-term memory; STADP (stage B), Executive functions / language; STADP (stage C), Episodic memory; VF, Test semantic Verbal Fluency.

* Correlation significantly at level 0.05 (bilateral);

** Correlation significantly at level 0.01 (bilateral).

The total score on the STADP, its three stages and the recognition of proverbs were correlated with the MMSE, semantic VF and CDT.

The highest correlation with the MMSE was seen with the total score of the STADP(r=0.64), followed by stage “C” – episodic memory (r=0.62) and by stage A – short-term memory (r=0.52); With the evocation of the words of MMSE, the strongest correlations were with the stage C (r=0.51) and total score (r=0.51) of the STADP. The stage B – executive functions and language – correlated better with semantic FV (r=0.47). The CDT had better correlation with stage A followed by stages C and B.

The highest correlation among these tests was between STADP total score and stage C (r=0.88), stage A (r=0.82) and stage B (r=0.59).

## Discussion

The performance of the patients with AD was lower than that of the control individuals on the MMSE, CDT and semantic VF as well as on the STADP. Only stage B (executive functions and language of the STADP, which assesses executive functions and language by means of the interpretation of proverbs) was unable to differentiate the groups. There is consensus that memory and executive function tests are the most appropriate tests for the diagnosis of AD.^6,20^ There is the possibility that the sample size of AD patients may have contributed toward minimizing the difference found. In addition, previous knowledge of the proverbs may have positively influenced the accuracy of their interpretation by the participants, including subjects with AD.

Concerning the STADP and usual screening tests, the best correlation was found with the MMSE^21^ (screening test of overall mental status). However, stage B of the STADP showed the best correlation with VF (a test suggested for executive functions and language)^11^ corroborating the findings in the literature. Semantic VF also showed good correlation with the whole of STADP. The CDT, used to evaluate executive function, showed a significant although not strong correlation with stage B of the STADP (r=0.29). These findings indicate that the STADP may have good internal consistency and is a test able to evaluate several cognitive domains.

The total score of STADP and stages A (short memory), C (episodic memory) and the recognition of the proverbs were inversely correlated with age. The mean age was higher in the group with AD than in the control individuals which may have contributed to our findings. However, stage B of the STADP, which assesses executive functions and language by means of the interpretation of proverbs, was not correlated with age.

The means of years of schooling were similar between the groups, greater than nine years, further increasing the reliability of our findings. Banhato and Nascimento^20^ previously demonstrated the existence of a link between formal education, understanding, and intellectual performance, besides identifying a trend of effect of schooling on tasks of abstract reasoning.

According to Ortiz and Bertolucci,^22^ in the initial stage of AD, language processing probably suffers interference from the decline in memory making it difficult to understand sentences, in agreement with findings of Mansur et al.^2^ that pointed to controversial issues regarding the possible factors contributing to this difficulty. The reduced storage capacity of short-term memory may be involved^2^. In our study, a significant correlation (r=0.48) was found between stage B (interpretation of the proverbs) and stage A (short-term memory). Other cognitive deficits may contribute to the language processing in early AD, such as problems in the central executive working memory as well as multi-factorial disorders (semantic aspects and effect processing). The literature affirms that there are correlations between memory, executive functions^21^ and language, which are the cognitive functions impaired in early AD.^2,6,19,23^ Moreover, the variable “proverb recognition” of the STADP showed a significant correlation (r=0.40) with stage A (short-term memory), indicating memory codification difficulties in the AD patients, a hypothesis in agreement with Hamdan and Bueno^6^ who evidenced impairment of storage in short-term memory in AD.

Regarding total score of STADP, the observed correlations with short-term and episodic memories, with executive functions and language, and the “recognition of proverbs” were also significant, suggesting that STADP can adequately evaluate cognitive deficits proposed. As Charchat et al.^1^ reported, the main neuropsychological characteristics of the early stages of AD are the impairment in episodic memory observed across all tests, more markedly in the tests of verbal memory, indicating a bilateral involvement of the temporal lobes, with a predominance of the dominant hemisphere (left), and the impairment of short-term memory which has been correlated with dysfunction in the frontal lobe.

The main limitation of this ongoing study is the small sample of AD patients. Notwithstanding, it was possible to verify that the STADP may prove a useful test for the diagnosis and evaluation of cognitive abilities in AD. Further studies comparing the STADP with other standardized tests, together with a better understanding of the cognitive deficits of early AD may contribute to screening of the disease, attributing to this instrument the proper contextualization proposed by Alchieri.^24^

## References

[1] Charchat H, Nitrini R, Caramelli P, Sameshima K (2001). Investigação de marcadores clínico dos
estágios iniciais da doença de Alzheimer com testes
neuropsicológicos computadorizados. Psicol Refl Crít.

[2] Mansur LL, Carthery MT, Caramelli P, Nitrini R (2005). Linguagem e cognição na doença de
Alzheimer. Psicol Refl Crít.

[3] Baddeley AD, Hitch G, Bower GA (1974). Working memory. The psychology of learning and motivation.

[4] Tulving E, Tulving E, Donaldson W (1972). Episodic and semantic memory. Organization of memory.

[5] Jacobson MW, Delis DC, Bondi MW, Salmon DP (2002). Do neuropsychological tests detect preclinical Alzheimer's
disease: individual test versus cognitive discrepancy score
analyses. Neuropsychology.

[6] Hamdan AC, Bueno O (2005). Relações entre controle executivo e memória
episódica verbal no comprometimento cognitivo leve e na
demência do tipo Alzheimer. Estud Psicol (Natal).

[7] Diniz CMC, Carvalho FRC, Minett TSC, Bueno OFA, Bertolucci PHF (2007). The assessment of executive functions in elderly with Alzheimer's
disease: clinical and functional correlations. Dement Neuropsychol.

[8] Lam LC, Lui VW, Chiu HF, Chan SS, Tam CW (2005). Executive function impairment in community elderly subjects with
questionable dementia. Dement Geriatr Cogn Disord.

[9] Fischer JS, Hannay HJ, Loring DW, Lezak MR, Lezak MD, Howieson DE, Loring DW (2004). Observational Methods, Rating Scales, and
Inventories. Neuropsychological Assessment.

[10] Okamoto IH (2001). Aspectos cognitivos da doença de Alzheimer no teste do
relógio: Avaliação de amostra da
população brasileira.

[11] Nitrini R, Caramelli P, Bottino C (2005). Diagnóstico de Alzheimer no Brasil:
avaliação cognitiva e funcional. Recomendações
do Departamento Científico de Neurologia Cognitiva e do
Envelhecimento da Academia Brasileira de Neurologia. Arq Neuropsiquiatr.

[12] Moretti R, Torre P, Antonello RM, Kazzato G, Bava A (2002). Ten-point clock test: a correlation analysis with other
neuropsychological tests in dementia. Int J Geriatr Psychiatry.

[13] Lacerda RC, Lacerda HRC, Abreu FS (1999). Dicionário de Provérbios. Francês,
Português, Inglês.

[14] Elmore CM, Gorhan DR (1957). Measuring the impairment of the abstracting function with the
proverbs test. J Clin Psychol.

[15] Silva CBP, Lomônaco JFB (1995). Elaboração e validação de um
instrumento para avaliar tipos de pensamento através da
interpretação de provérbios. Psic.: Teor Pesq.

[16] Lomônaco JFB, Claro ECF, Sousa JTP, Mori NNR, Barrera SD, Lima VS (1995). Escolaridade e capacidade de abstração: um estudo
com Teste Brasileiro de Provérbios. Psic.: Teor Pesq.

[17] Siviero MO (1997). Capacidade de abstração e o Teste de
Provérbios.

[18] American Psychological Association (1998). Presidential task force on the assessment of age-consistent
memory decline and dementia: guidelines for the evaluation of dementia and
age-related cognitive decline. Am Psychol.

[19] Santos MTF, Carvalho TL, Bastos O, Sougey EB (2005). Estudo piloto de desempenho mnêmico com "Jogo de
Memória de Provérbios" criado para idosos. Neurobiologia.

[20] Banhato EFC, Nascimento E (2007). Funções executivas em idosos: um estudo utilizando
subtestes da Escala WAIS-III. Psico-USF.

[21] Folstein MF, Folstein SE, McHugh PR (1975). "Mini-Mental State". A practical method for grading the cognitive
state of patients for the clinician. J Psychiatr Res.

[22] Ortiz KZ, Bertolucci (2005). Alterações de linguagem nas fases iniciais da
doença de Alzheimer. São Paulo. Arq Neuropsiquiatr.

[23] Bäckman L, Jones S, Berger A-K, Laukka EJ, Small BJ (2005). Cognitive impairment in preclinical Alzheimer's disease: a
meta-analysis. University of South Florida Neuropsychology.

[24] Alchieri JC, Andrade VM, Santos FH, Bueno OFA (2004). Aspectos instrumentais e metodológicos da
avaliação psicológica. Neuropsicologia Hoje.

